# Mechanical Properties and Potential Clinical Implications of Improved Superelastic Orthodontic Archwires: An Observational Study

**DOI:** 10.7759/cureus.48334

**Published:** 2023-11-05

**Authors:** Dilip Srinivasan, Raj Kumar Krishnan

**Affiliations:** 1 Orthodontics and Dentofacial Orthopaedics, SRM Dental College and Hospital, Chennai, IND; 2 Oral and Maxillofacial Pathology, SRM Dental College and Hospital, Chennai, IND

**Keywords:** brackets, orthodontic force, niti, cuniti, super elastic, arch wires

## Abstract

Background: Superelastic materials have gained popularity due to their ability to maintain a constant force over a prolonged period during orthodontic treatment. However, high hysteresis and frictional properties had limited the use of superelastics as archwire material that demanded the need for improved superelastic orthodontic archwires with enhanced mechanical properties.

Aim: The present study aimed to investigate the differences in mechanical properties and frictional resistance of improved superelastic orthodontic archwires against conventional archwires and to evaluate their potential implications in clinical orthodontic practice.

Materials and methods: A total of 45 samples with 15 in each category respectively from low hysteresis superelastic archwire (L&H Titan; Tomy Inc., Tokyo, Japan), nickel-titanium (NiTi) archwires (Ormco, Brea, CA, USA) and NiTi with copper (CuNiTi) archwires (Ormco) of equal diameter (0.016 x .022 inches) and length (10 cm) were randomly assigned in combination among metal and ceramic orthodontic brackets group. The frictional properties of the archwires were measured using a universal testing machine (Instron, Norwood, MA, USA) equipped with a custom-made jig. The load-displacement data were recorded, and other mechanical properties that included tensile strength, compressive strength and deflective force at 4mm were also evaluated. The data were analysed using independent Student t-tests to compare the mean frictional resistance of the three archwires followed by analysis of variance (ANOVA) to evaluate differences between the means with p-value of less than 0.05 considered as statistically significant.

Results: The improved superelastic wires had the least frictional resistance among the three archwires tested. Further intergroup comparison to evaluate differences between the frictional resistance means among the three archwire categories with two orthodontic brackets groups revealed a significant difference at p<.05. Pairwise comparison also showed significant differences with higher frictional resistance between metal brackets and low hysteresis superelastic archwire category than ceramic brackets and NiTi with copper archwires (.0003) and ceramic brackets with NiTi archwires category (.003) respectively. The lowest deflective force at 4mm with better tensile and compressive strength was seen with the improved superelastic wires.

Conclusion: The results of this study suggest that low hysteresis superelastic archwires have lower frictional forces when combined with metal orthodontic brackets compared with ceramic orthodontic brackets. Better tensile strength with least compressive strength and deflective forces at 4mm of testing among low hysteresis L&H Titan superelastic archwire than CuNiTi and NiTi archwires was observed making them potentially advantageous for orthodontic applications.

## Introduction

Orthodontic treatment is a field of dentistry that aims to improve the occlusion and appearance of teeth by correcting their alignment and position. One of the most important components in orthodontic treatment is the archwire, which applies force to the teeth and guides them to their correct position. Over the years, a wide range of materials has been used for orthodontic archwires, such as stainless steel (SS), beta-titanium, and nickel-titanium (NiTi). Among these materials, NiTi has emerged as a popular choice due to its unique properties such as shape memory and superelasticity, which enable it to apply a continuous and gentle force to the teeth, resulting in faster and more comfortable treatment [1,2].

NiTi archwires are known for their superelasticity, which is the ability to recover their original shape after being deformed and maintain a constant force over a prolonged period [3]. This unique property makes them ideal for orthodontic applications, as they can apply a consistent force to the teeth without permanent deformation or loss of mechanical properties [4]. However, the superelastic behaviour of NiTi archwires is also associated with a phenomenon known as hysteresis, which is the energy loss that occurs during deformation and recovery. Hysteresis can affect the clinical performance of the archwire, as it can cause the wire to lose its shape memory and superelasticity over time, leading to reduced treatment efficiency and increased discomfort for the patient. To address the issue of hysteresis in NiTi archwires, researchers have developed low hysteresis NiTi alloys, which exhibit reduced energy loss during deformation and recovery [5,6].

On the other hand, the frictional properties of archwires are also an important consideration in orthodontic treatment as they affect the amount of force required to move the tooth. Friction is the force that opposes the movement of the wire through the bracket, and it can cause increased discomfort for the patient and reduced treatment speed. Friction can occur between the archwire and the brackets causing unwanted tooth movement or slowing down the rate of tooth movement thus bringing about untoward or ineffective orthodontic treatment [7]. Despite recent recommendations on the use of low hysteresis NiTi archwires, the frictional properties of low hysteresis NiTi archwires have not been studied in detail in the literature. Recent literature studies have also shown low hysteresis NiTi archwires may provide greater comfort and reduced treatment time for patients. It was evident that these alloys have the potential to improve the clinical performance of NiTi archwires by maintaining their shape memory and superelasticity over an extended period of time [8]. Nonetheless, the potential nature of these low hysteresis NiTi archwires for reducing friction and improving treatment efficiency still remains unclear. Hence this study aims to investigate the differences in frictional resistance properties of improved superelastic orthodontic archwires (ISW) against conventional archwires and to evaluate their mechanical property with potential implications in clinical orthodontic practice.

## Materials and methods

**Figure 1:**
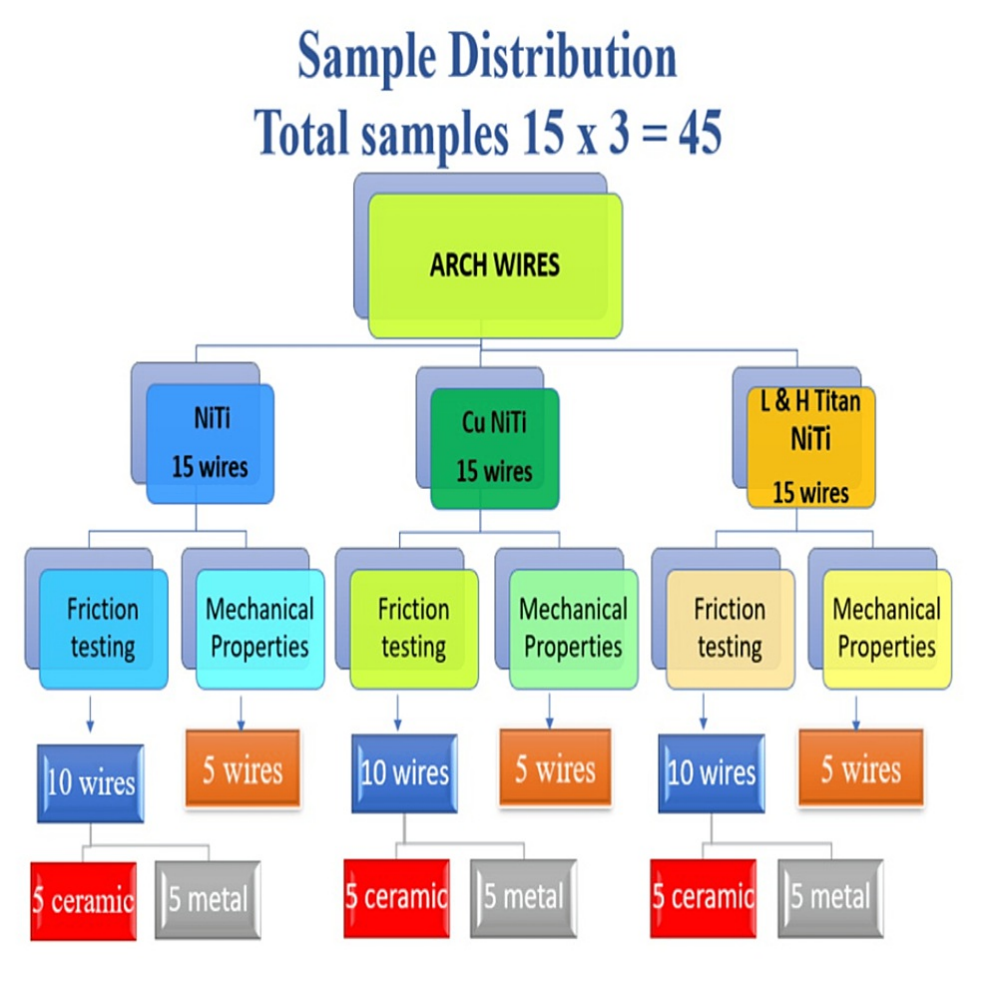
Study Sample Distribution. NiTi: nickel-titanium, CuNiTi: copper nickel-titanium

A total of 45 samples with 15 in each category (Figure 1) respectively from low hysteresis superelastic archwires (L&H Titan; Tomy Inc., Tokyo, Japan), NiTi archwires (Ormco, Brea, CA, USA) and NiTi with copper (CuNiTi) archwires (Ormco) of equal diameter (0.016 x .022 inches) and length (10cm) were randomly assigned in combination among metal and ceramic orthodontic brackets group. In every archwire category, frictional testing was carried out on 10 samples distributed among metal and ceramic orthodontic brackets groups of five each while the remaining five samples were used for assessing mechanical properties.

**Figure 2:**
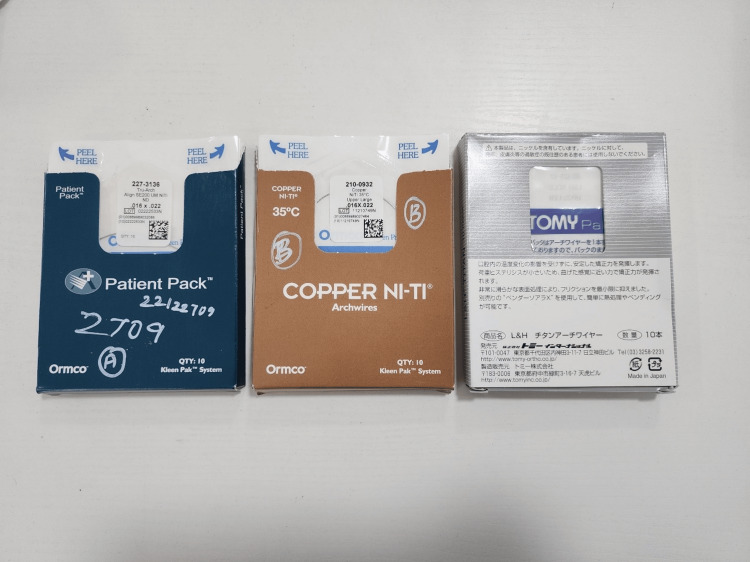
Various archwires used in the study. nickel-titanium (NiTi) and copper NiTi: Ormco; L&H Titan: Tomy Inc.

**Figure 3:**
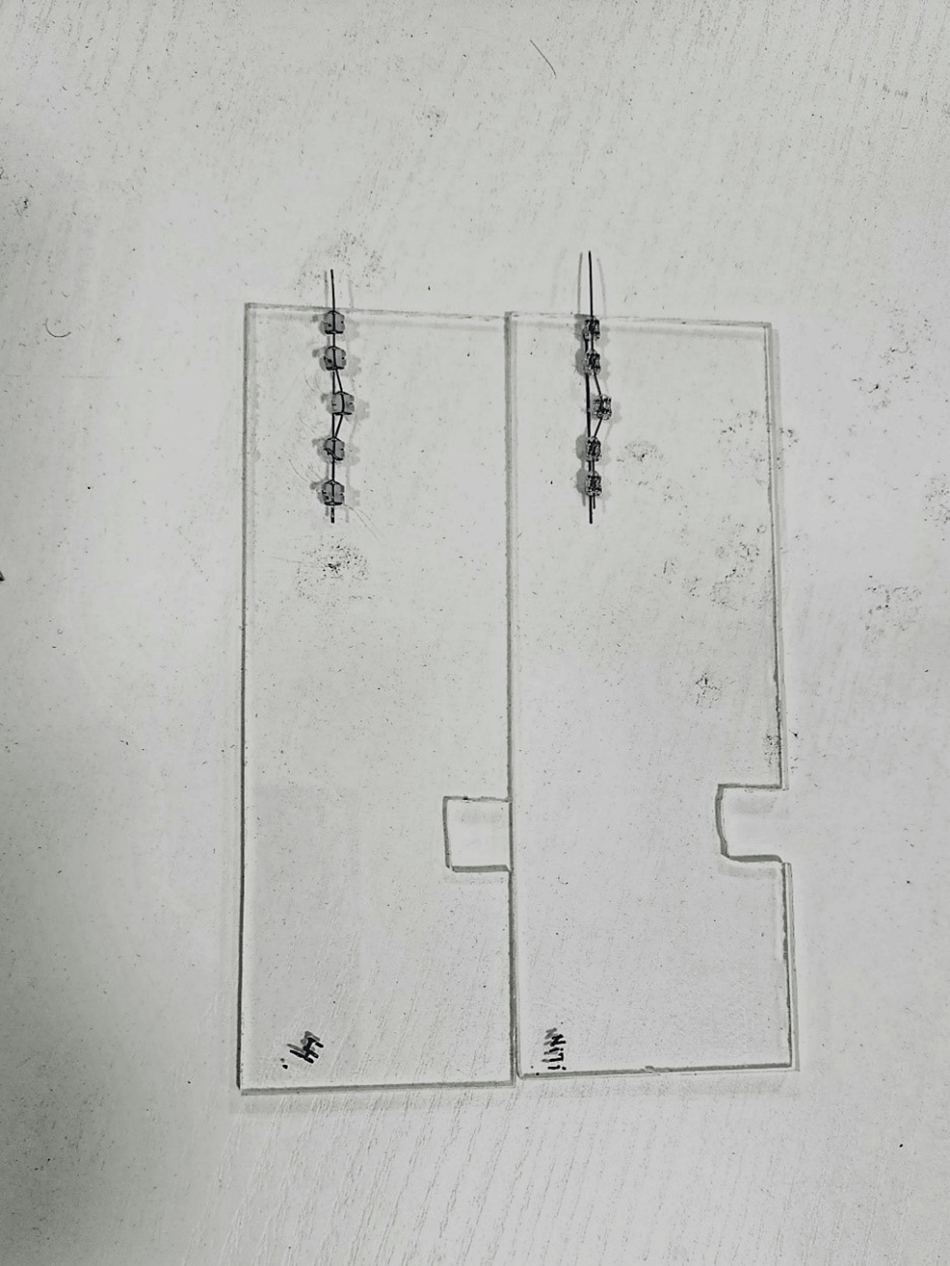
Custom-made jig-saw for friction testing (right side).

Prior to testing, the archwires were cleaned using isopropyl alcohol and dried with compressed air. The frictional properties of the archwires were measured using the universal testing machine (Instron, Norwood, MA, USA) equipped with a custom-made jig. The jig consisted of five brackets with the archwire passing through the brackets and secured by ligature wire. A 50g load was applied to each archwire and the frictional force was measured as the archwire was pulled through the brackets at the rate of 0.5mm/min (Figures 2, 3) [9,10]. Institutional ethical clearance was obtained (SRMDC/IRB/2018/PhD/No.102).

**Figure 4:**
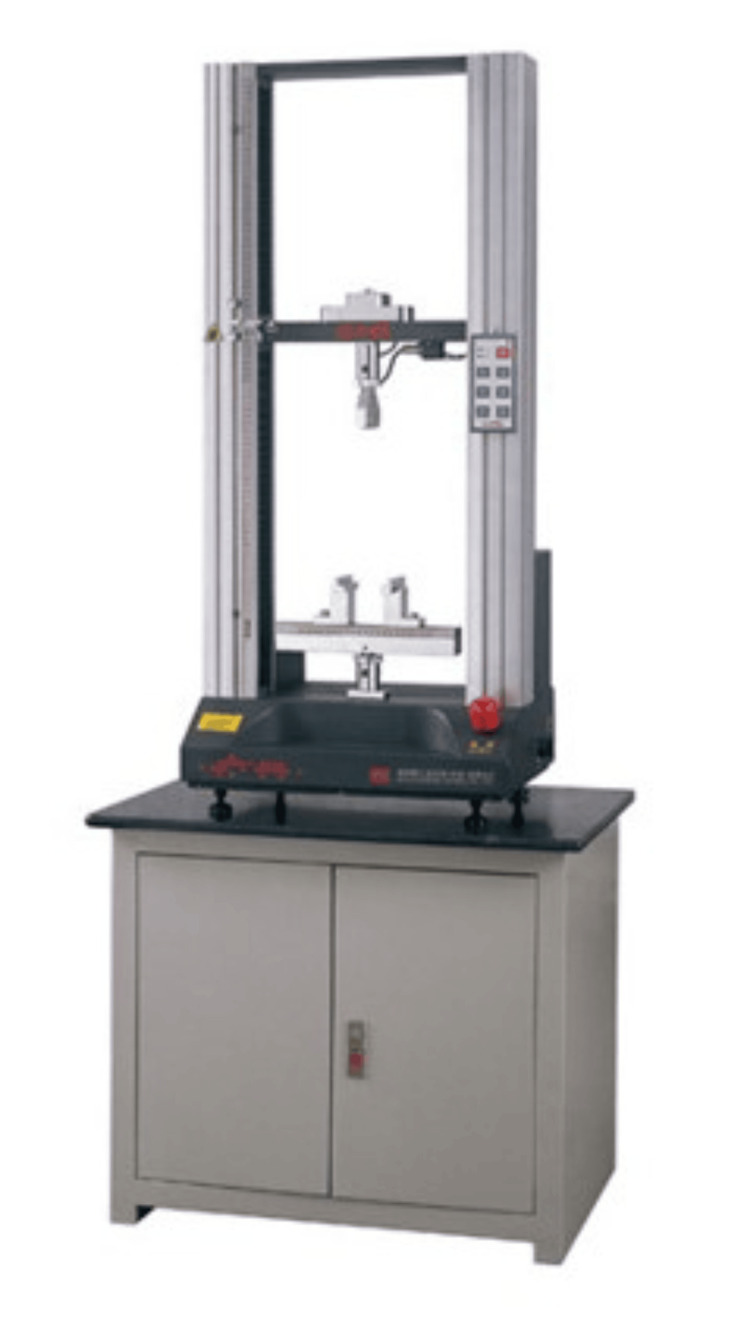
Universal testing machine (Instron).

The mechanical properties of the archwires were also measured using the universal testing machine (Figure 4). The archwire was secured in the machine's grips, and a tensile load was applied at a rate of 1mm/min until the wire fractured. The load-displacement data were recorded, and other mechanical properties that included tensile strength, compressive strength and deflective force at 4mm were also calculated. Independent Student t-tests were used to compare the mean frictional resistance between metal and ceramic orthodontic brackets group. Kruskal-Wallis test followed by Dunns post-hoc test was performed to compare the frictional resistance between the three archwire categories while one-way ANOVA and Tukey’s honest significant difference (HSD) was done to evaluate the differences in the tensile strength, compressive strength and deflective force between the archwires with an overall p-value of less than 0.05 considered as statistically significant.

## Results

A descriptive analysis of the results was performed to evaluate the frictional resistance mean and standard deviation of metal and ceramic orthodontic bracket groups. The metal group displayed a mean frictional force of 5.8287 ± 2.98202 Newton (N) (mean ± S.D) while the ceramic group showed 11.3656 ± 3.4218 N (mean ± S.D). Independent t-test revealed significant differences in the force exerted with the ceramic group showing a higher mean value than the metal group (p<.05) (Table 1).

**Table 1:** Showing the frictional resistance mean and standard deviation of metal and ceramic orthodontic bracket group. *p<.05: Statistically significant

Newton	mean	Standard deviation	Equal variances	Sig. (2-tailed)	Mean difference	Std. error difference	95% confidence interval
Metal	5.8287	2.98202	Equal variances assumed	.000*	-5.53694	1.171945	-7.9375
Ceramic	11.3656	3.42189	Equal variances not assumed	.000*	-3.13429	1.060834	-5.8264

Independent Student t-test for friction resistance showed a mean frictional force of 6.26555 ± 2.03444 N (mean ± S.D) in the metal group and 13.8499 ± 4.050 N (mean ± S.D) in the ceramic group among the NiTi archwires category and mean frictional force of 7.5487 ± 4.1229 N (mean ± S.D) in the metal group and 10.87514 ± 3.2514 N (mean ± S.D) in the ceramic group among the CuNiTi archwires category while the low hysteresis L&H Titan superelastic archwire category revealed a mean frictional force of 3.67187 ± 0.48387 N (mean ± S.D) in the metal group and 9.3718 ± 1.0116 N (mean ± S.D) in the ceramic group respectively.

One-way ANOVA analysis showed no significant differences between and within the metal orthodontic group among the three archwire categories (Table 2).

**Table 2:** Showing the one-way ANOVA between and within the metal orthodontic brackets groups. *p<.05: Statistically significant

Newton	Sum of squares	df	Mean square	F	Sig
Between the groups	39.007	2	19.503	2.738	.105
Within the groups	85.488	12	7.124	-	-
Total	124.495	14	-	-	-

**Figure 5:**
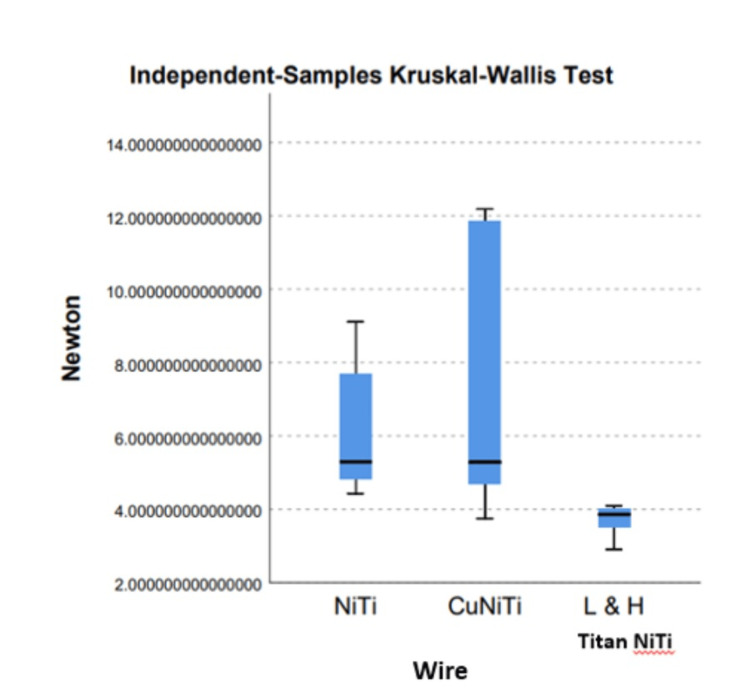
Independent-samples Kruskal-Wallis test. NiTi: nickel-titanium, CuNiTi: copper nickel-titanium

Kruskal-Wallis test performed to compare the frictional resistance between the three archwire categories showed a significant difference of .026 with lower frictional forces among the low hysteresis superelastic archwires (Figure 5).

Pairwise comparison of wire also revealed significant differences between the low hysteresis L&H Titan superelastic archwire category with NiTi and CuNiTi archwires (.024) and NiTi archwires category (.016) respectively (Table 3).

**Table 3:** Showing the pairwise comparison of wire within the metal orthodontic brackets groups. Each row tests the null hypothesis that the Sample 1 and Sample 2 distributions are the same. a- Significance values have been adjusted by the Bonferroni correction a. for multiple tests. *p<.05: Statistically significant NiTi: nickel-titanium, CuNiTi: copper nickel-titanium

Sample-1- Sample 2	Test statistic	Std. error	Std. test statistic	sig	Adj. sig ^a^
L&H Titan NiTi-CuNiTi	6.4000	2.828	2.263	.024*	.071
L&H Titan NiTi-NiTi	6.8000	2.828	2.404	.016*	.049
CuNiTi-NiTi	.400	2.828	.141	.888	1.000

**Figure 6:**
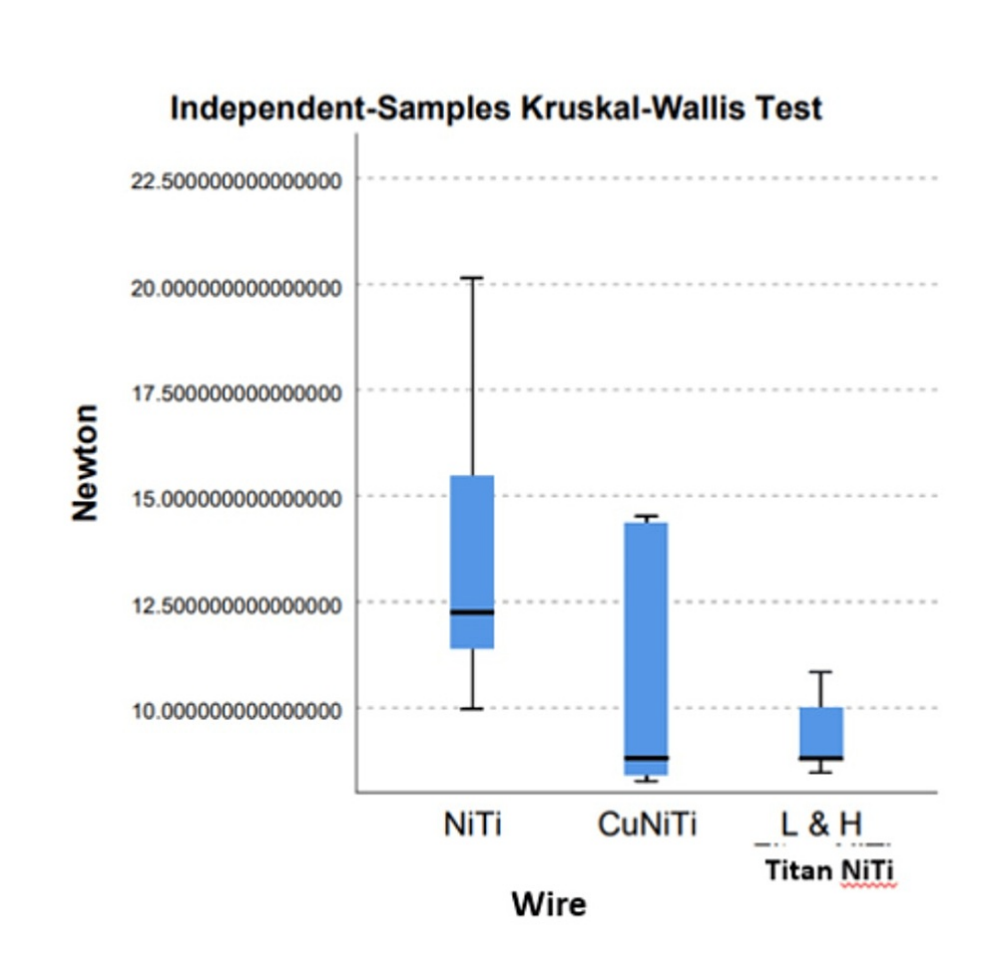
Independent-samples Kruskal-Wallis test. NiTi: nickel-titanium, CuNiTi: copper nickel-titanium

One-way ANOVA analysis showed no significant differences between and within the metal orthodontic group among the three archwire categories (Table 4, Figure 6). Kruskal-Wallis test performed to compare the frictional resistance between the three archwire categories also showed no significant difference (.102) however slight differences in the frictional forces were observed among the low hysteresis superelastic archwires.

**Table 4:** Showing the one-way ANOVA between and within the ceramic orthodontic brackets groups. *p<.05: Statistically significant

Newton	Sum of squares	Df	Mean square	F	Sig
Between the groups	51.938	2	25.969	2.783	.102
Within the groups	111.993	12	9.333	-	-
total	163.931	14	-	-	-

Further intergroup comparison and one-way ANOVA analysis to evaluate differences between the means among the three archwire categories with two orthodontic brackets groups revealed a significant difference at p<.05 (Table 5).

**Table 5:** Showing the one-way ANOVA between and within the metal and ceramic orthodontic brackets groups among three archwire categories. NiTi: nickel-titanium, CuNiTi: copper nickel-titanium

Newton	Samples	Sum of squares	df	Mean squares	F	Sig
Between the Groups	Metal + NiTi Metal + CuNiTi Metal + L&H Titan NiTi Ceramic + NiTi Ceramic + CuNiTi Ceramic + L&H Titan NiTi	320.878	5	64.176	7.799	.000*
Within the Groups	Metal + NiTi Metal + CuNiTi Metal + L&H Titan NiTi Ceramic + NiTi Ceramic + CuNiTi Ceramic + L&H Titan NiTi	197.481	24	8.228	-	-
Total	-	518.359	29	-	-	-

One-way ANOVA followed by post-hoc Tukey’s HSD showed significant differences between metal brackets and low hysteresis L&H Titan superelastic archwire category with ceramic brackets and CuNiTi archwires (.000), ceramic brackets with NiTi archwires category (.007) respectively. Kruskal-Wallis test performed to compare the frictional resistance between the three archwire categories showed a significant difference of .002 with lower frictional forces among the metal brackets with low hysteresis superelastic archwires.

**Figure 7:**
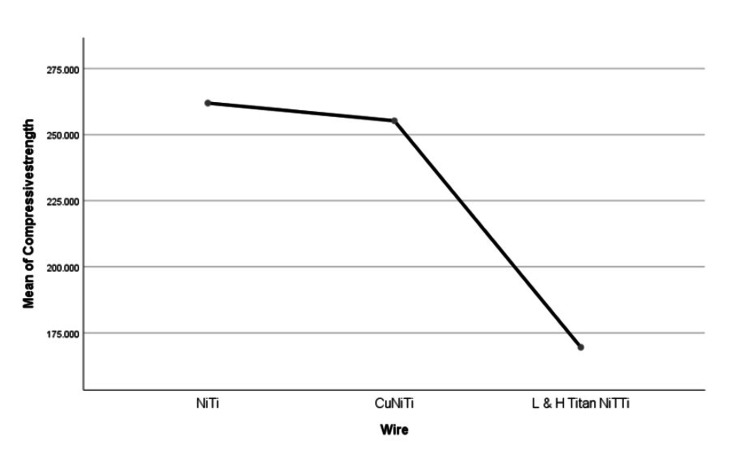
Image of deflective force at 4mm. NiTi: nickel-titanium, CuNiTi: copper nickel-titanium

Pairwise comparison of wire also revealed significant differences between metal brackets and low hysteresis L&H Titan superelastic archwire category than ceramic brackets and CuNiTi archwires (.003) and ceramic brackets with NiTi archwires category (.000) respectively. One-way ANOVA and Tukey’s HSD were done to evaluate the differences in the compressive strength and deflective force at 4mm of the testing showed a mean compressive strength of 261.943 ± 41.9376 (mean ± S.D) among NiTi archwires, 255.235 ± 28.6017 (mean ± S.D) among CuNiTi archwires and 169.527 ± 23.5950 (mean ± S.D) among low hysteresis L&H Titan superelastic archwires respectively. Post-hoc Tukey’s HSD revealed significant differences between the means (p<.05) (Table 6, Figure 7) with a lower compressive strength and least deflective forces at 4mm of testing among the low hysteresis L&H Titan superelastic archwire category.

**Table 6:** Showing the one-way ANOVA of compressive strength between and within the three archwire categories.

Newton	Sum of squares	df	Mean square	F	Sig
Between the groups	26552.336	2	13276.168	12.710	.001*
Within the groups	12534.207	12	1044.517	-	-
total	39086.543	14	-	-	-

**Figure 8:**
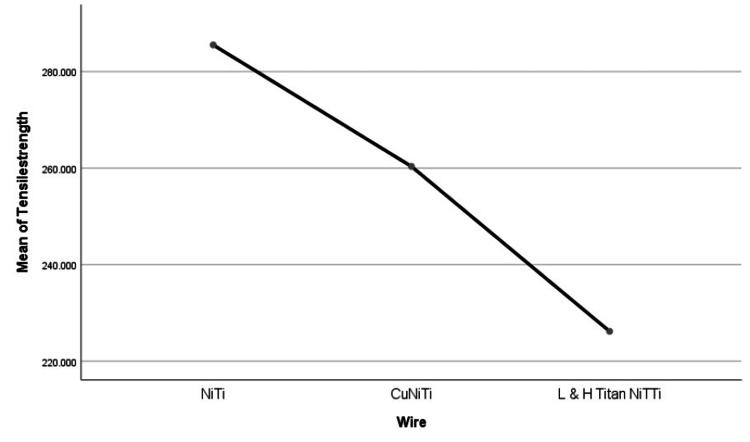
Tensile strength mean plots. NiTi: nickel-titanium, CuNiTi: copper nickel-titanium

One-way ANOVA and Tukey’s HSD was done to evaluate the differences in the tensile strength showed a mean compressive strength of 285.522 ± 4.041 (mean ± S.D) among NiTi archwires, 260.344 ± 36.972 (mean ± S.D) among CuNiTi archwires and 226.175 ± 12.5983 (mean ± S.D) among low hysteresis L&H Titan superelastic archwires respectively. Post-hoc Tukey’s HSD revealed significant differences between the means (p<.05) (Table 7, Figure 8) with a lower tensile strength among the low hysteresis L&H Titan superelastic archwire category.

**Table 7:** One-way ANOVA of tensile strength between and within the three archwire categories.

Newton	Sum of squares	df	Mean square	F	Sig
Between the groups	8872.593	2	4436.297	8.631	.005*
Within the groups	6167.990	12	513.999	-	-
total	15040.584	14	-	-	-

## Discussion

Superelastic materials have gained popularity due to their ability to maintain a constant force over a prolonged period during orthodontic treatment. However, one of the major disadvantages is their high hysteresis and frictional properties which can lead to loss of force over time and has limited the use of superelastics as archwire material that demanded the need for improved superelastic orthodontic archwires with enhanced mechanical properties. Hence the present study was carried out to evaluate the differences in mechanical properties and frictional resistance of improved superelastic orthodontic archwires.

Katz et al. in an observation study showed descending a tooth along an archwire during the course of an orthodontic treatment involves a frictional type of force that resists this tooth movement that can cause several adverse effects and recommends the need for a self-lubricating substance with NiTi wires or CuNiTi wires or any improved material as used in the present study to reduce this effect [11]. The present study showed the highest friction with NiTi wires followed by CuNiTi wires and the least with the improved superelastic wires when using ceramic brackets while the highest friction for metal brackets was seen with CuNiTi wires. Pairwise comparison also revealed significant differences with lowest friction or higher frictional resistance between metal brackets and the low hysteresis superelastic archwire category than ceramic brackets and NiTi with copper archwires (.0003) and ceramic brackets with NiTi archwires category (.003) respectively. Liaw et al. in a similar study on the frictional behaviour of an improved superelastic NiTi wire with low hysteresis also showed narrower stress hysteresis and less friction with improved mechanical properties [12]. Bednar et al. in a comparative study on frictional forces, resistance between the orthodontic brackets and archwires noted that elastomeric-ligated ceramic brackets had the highest friction when compared to other methods of ligation. They also felt that ceramic brackets would cause more anchorage strain than stainless steel brackets [13]. Our study results were also in agreement with the above observations and had also shown increased friction in all archwire categories among the ceramic brackets group compared to metal brackets.

Yonemitsu et al. in a clinical case observation on characters and clinical application of improved superelastic NiTi alloy archwires demonstrated the use of low hysteresis superelastic archwire in all the stages of orthodontic treatment except cases requiring heavy forces following orthognathic surgeries [14]. The study also witnessed the material to be effective during levelling stage in uprighting molars, close spaces using tipping movements by manually bending the wire thus increasing the stiffness of the wires, the elastic modulus of the bent region with control force and movement owing to their superelasticity, shape-memory effect, and low hysteresis. The present study showed mean deflective force was highest with NiTi wires and lowest with the improved superelastic wires when tested at 4mm. Post-hoc Tukey’s HSD also revealed significant differences between the means (p<.05) with a lower compressive strength and least deflective forces at 4mm of testing among the low hysteresis L&H Titan superelastic archwires category. Ishida et al. in a non-growing skeletal class II malocclusion patient used improved superelastic NiTi archwires in combination with coil springs and established constant and continuous low force by improved superelastics to the dentition [15]. This result suggests that low hysteresis archwires could lead to faster and more efficient tooth movement during orthodontic treatment due to their reduced frictional resistance and low deflective force over a large deflection.

Limitations of the study

Extensive physico-chemical material characterization can be included in the study. Sample size can be increased and titanium molybdenum alloy (TMA) or SS wire can also be used to compare with the study groups.

## Conclusions

Orthodontic treatment is a field of dentistry that involves the use of various types of wires to guide teeth movement. Among the different types of wires used, superelastic archwires have gained popularity due to their ability to maintain a constant force over a prolonged period. Frictional properties of archwires are an important consideration in orthodontic treatment as they affect the amount of force required to move teeth. Friction can occur between the archwire and the brackets, causing unwanted tooth movement or slowing down the rate of tooth movement. The results showed that the highest friction was seen with NiTi wires followed by CuNiTi wires and least with the Improved superelastic wires when using ceramic brackets. The highest friction for metal brackets was seen with CuNiTi wires. The result was found to be statistically significant between ISW and the other two archwires. Ceramic brackets showed increased friction in all wires compared to metal brackets. The deflective force was highest with NiTi wires and lowest with the improved superelastic wires when tested at 4mm. This result suggests that low hysteresis archwires could lead to faster and more efficient tooth movement during orthodontic treatment due to their reduced frictional resistance and low deflective force over a large deflection.
